# Biodeterioration and Chemical Corrosion of Concrete in the Marine Environment: Too Complex for Prediction

**DOI:** 10.3390/microorganisms11102438

**Published:** 2023-09-28

**Authors:** Christine C. Gaylarde, Benjamin Otto Ortega-Morales

**Affiliations:** 1 Department of Microbiology and Plant Biology, University of Oklahoma, 770 Van Vleet Oval, Norman, OK 73019, USA; 2 Center of Environmental Microbiology and Biotechnology, Universidad Autónoma de Campeche, Av. Agustín Melgar s/n entre Juan de la Barrera y Calle 20, Col. Buenavista, San Francisco de Campeche, Campeche 24039, Mexico; beortega@uacam.mx

**Keywords:** biofilm, colonization, degradation, microorganisms, subtidal, intertidal and splash zones, extracellular polymeric materials (EPS)

## Abstract

Concrete is the most utilized construction material worldwide. In the marine environment, it is subject to chemical degradation through reactions with chloride (the most important ion), and sulfate and magnesium ions in seawater, and to biodeterioration resulting from biological (initially microbiological) activities, principally acid production. These two types of corrosions are reviewed and the failure of attempts to predict the degree of deterioration resulting from each is noted. Chemical (abiotic) corrosion is greatest in the splash zone of coastal constructions, while phenomenological evidence suggests that biodeterioration is greatest in tidal zones. There have been no comparative experiments to determine the rates and types of microbial biofilm formation in these zones. Both chemical and microbiological concrete deteriorations are complex and have not been successfully modeled. The interaction between abiotic corrosion and biofilm formation is considered. EPS can maintain surface hydration, potentially reducing abiotic corrosion. The early marine biofilm contains relatively specific bacterial colonizers, including cyanobacteria and proteobacteria; these change over time, producing a generic concrete biofilm, but the adhesion of microorganisms to concrete in the oceans has been little investigated. The colonization of artificial reefs is briefly discussed. Concrete appears to be a relatively prescriptive substrate, with modifications necessary to increase colonization for the required goal of increasing biological diversity.

## 1. Introduction

### History and Functions of Concrete

Concrete is an artificial (consolidated) material, with properties similar to natural stone. It is a constructional material made from a mixture of cement, fine aggregates (usually sand), coarse aggregates (gravel or crushed rock), and water. The mixture hardens (cures) with time to produce a strong, durable, reflective, and versatile material that is employed in huge quantities for construction worldwide. A form of concrete has been in use for thousands of years. The first proven concrete structure was the floor of a hut in what is modern-day Israel, made about 7000 BC by burning limestone to produce quicklime that was then mixed with stone and water before leaving to solidify (The Irish Concrete Society, https://concrete.ie.about>concrete, accessed 2 May 2023). Portland cement was invented in England in 1824; it consists of four main compounds: tricalcium silicate, dicalcium silicate, tricalcium aluminate, and tetra-calcium aluminoferrite. This innovation led to the subsequent developments that have made concrete, without doubt, the most important building material worldwide [1]. A full and fascinating history can be found in [2].

The internal structure of concrete, with its complicated pore structure, is determined by the cement hydration reaction, which is complex. Studies to determine the structures of the many varieties of concrete have involved the use of, for example, synchrotron X-rays [3] and neutron scattering [4]. Pore structure has generally been determined by mercury intrusion porosimetry (e.g., [5]). Recent developments for improving some of the properties of concrete (such as drying shrinkage, crack resistance, and freeze–thaw durability) include the addition of fly ash [6], glass fiber [7], and nanoparticles [8]. Most recently, the use of old concrete, as recycled aggregate, has come to the fore [9], reducing the need for natural aggregates, whose use is becoming unsustainable [10].

Concrete, then, is employed all over the world in many different situations. This article concentrates on concrete used for marine structures. The development of the North Sea oil industry in the 1970s led to the construction of around 20 concrete platforms containing approximately 2 million m^3^ of high-quality concrete [11]. In addition, there are pipes, tunnels, underwater foundations (such as those of the famous Norwegian underwater restaurant “Under”), seawalls, storm barriers, breakwaters, revetments, and other deep sea and shoreline structures. Although the aim of this article is to review the negative effects of seawater chemistry and microorganisms on the structure of concrete, artificial reefs will also be considered briefly. These are structures where biological colonization is deemed desirable in order to encourage biodiversity; hence there has been more interest in their biocolonization.

## 2. The Marine Environment and Concrete Deterioration

The deterioration of concrete is still referred to, especially by engineers and materials scientists, as “corrosion”, although this term is defined in the Collins English Dictionary, Wikipedia, and other sources as a deterioration of metal that is associated with an electrochemical reaction with the environment. In this article, we shall use the alternatively accepted definition of corrosion that includes materials other than metals. 

The seawater environment is one of the most aggressive for concrete, because of its chloride, sulfate, and magnesium levels [12]. The aggressive nature of seawater is increased by biofouling and the growth of marine microorganisms and macroorganisms on the surface and within the concrete [13,14]. There is extensive literature on biofouling of concrete structures in the marine environment [15,16,17,18,19] and macrofouling (growth of higher organisms such as bivalves, algae, and seagrasses) will not be covered in the current article; they are outside the remit of this special issue. In the case of reinforced concrete, only the deterioration of the concrete component, and not the metal, will be reviewed. We will examine the interactions between built concrete structures and seawater under vertical (tidal) zonation and consider the associated marine microorganisms that initiate biodeterioration, which occurs together with non-biological activities such as carbonation and chloride and sulfate attack. Carbonation by carbon dioxide is necessary to reduce the initially high surface pH of new concrete to around 9, enabling further chemical reactions as well as the adhesion of microorganisms. 

### 2.1. Chemical Corrosion

Following carbonation, a severe chemical attack occurs in the presence of the surrounding chloride and sulfate ions in seawater. Chlorides can be incorporated in calcium chloroaluminate hydrates attached to calcium-silicate hydrate, blocking pores [20]. Sulfate ions react with cement hydration products to produce gypsum and ettringite, which damage the concrete matrix and increase chloride penetration [21]. The high chloride content of seawater, however, can suppress the formation of ettringite [22]. Magnesium is not corrosive at the normally low pH of seawater or on the carbonated surface but becomes more aggressive at the high pH levels (above pH 12) encountered within the concrete pores when brucite precipitates (12]. This ion, however, shows limited penetration into concrete, affecting only the surface layers [22]. 

The depth below the sea surface, governing oxygen and particulate levels in the water, influences the overall reaction rate, as does the exact exposure regime of the concrete to seawater. Vertical zonation defines intertidal and subtidal ecosystems. Subtidal ecosystems are always submerged, whereas intertidal ecosystems are found between the high and low (flood and ebb) tides, experiencing fluctuating influences of land, open air, and sea (triple interfaces). This contrasting exposure to the seawater column and atmosphere influences a differential chemical and physical regime in terms of salinity, oxygen, water stress (desiccation), etc. These chemical differences influence physicochemistry and atmospheric exposure, directly affecting the rate of concrete deterioration. Salinity, which varies according to climate and geographic conditions, obviously affects chloride ingress into the concrete, with resulting loss of compressive strength; however, other ions, such as sulfate and magnesium, can suppress the reaction with chloride [22].

The durability of concrete in marine environments differs depending on the seawater exposure regime: atmospheric, splash, tidal, or submerged [23]. The most damaging conditions are found in the splash zone [24,25], where chloride transport is greater, oxygen is readily available [26], and sulfate attack is more important [27]. The splashing action can remove previously formed, non-adhesive corrosion products, allowing them to re-form when the area is once again covered by the sea [22]. In addition, this region suffers from wave impact, freezing, and thawing in certain geographical locations, as well as wetting and drying cycles [22]. Where concrete has dried and possibly cracked during temporary open-air exposure in hot environments for example, the subsequent reimmersion can cause the hydration of products, leading to the formation of ettringite in the pores and cracks, with resulting loss of strength [27]. This tidal zone exposure is less aggressive than the splash zone, but generally more inducive of corrosion than the submerged zone with its relatively low oxygen levels. In the submerged zone, oxygen levels are limited and thus corrosion reactions are reduced [28]. Salt weathering is the only type of attack and there is no alleviation of the chemically-induced corrosion products. Ref. [29] confirmed the relative aggressivity of atmospheric, immersion, and splash marine zones for ordinary Portland cement (OPC) and two types of cement blended with OPC (PPC and PSC), exposed over 10 years. They reported that blended cement showed reduced resistance to chloride penetration and increased biofilm formation, but no explanation was given for these results.

Chloride is regarded as the most important abiotic influence on marine concrete corrosion. Although there are areas in which the chloride content of marine waters is low (e.g., the Baltic and Black Seas, at 3000 and 8500 mg·L^−1^, respectively), the main constituents of seawater around the Earth are similar. Impinging chloride ions rapidly produce a so-called “skin effect” on the immersed concrete surface where chloride concentrations are at their highest levels [30]. There have been many attempts to model the ingress of aggressive ions and subsequent deterioration reactions in concrete subjected to the marine environment [28,31,32,33]. Results differ considerably, making this a highly complex area.

### 2.2. Microbiological Corrosion (Biodeterioration)

The study of microbial deterioration of concrete involves materials science, mineralogy, and microbial ecology. Many authors have considered the corrosion of concrete in sewer pipes, and biodeterioration in these structures has been thoroughly investigated (see, for example, reviews [34,35,36]); however, information on marine biocorrosion of concrete is relatively scarce. Seawater composition and environmental parameters such as temperature, pH, and pollution influence not only the chemical corrosion of concrete but also adhering microorganisms. Alternating conditions of wetting and drying have been shown to increase chemical deterioration of poor-quality concrete in seawater [37]; whether microbially induced deterioration is also affected is unknown. The microbial species attaching to concrete structures that are permanently or intermittently submerged in seawater might be expected to differ, with intermittently submerged biofilms drying out periodically, favoring the more dehydration-resistant organisms. Conversely, the presence of a biofilm could maintain a degree of humidity on the concrete surface, reducing the drying effects over the exposure period. Controlled experiments to determine the effects of biofilm on the physicochemical corrosion of concrete have never been performed. Indeed, there is as yet no information on the microbiology of concrete subjected to the different sea immersion zones: splash, tidal, and submerged. 

The microbial content and abundance of the oceans differ substantially around the globe. Does the attached microbial population on concrete differ equally, or is there a specific concrete biofilm on marine structures? The recent interest in marine pollution by microplastics has led to the identification of a plastic-specific biofilm (the “plastisphere”, [38]); whether the equivalent exists for concrete (a concretosphere?) is unknown, although there is no evidence of this from studies on the better-studied sewer pipe corrosion. Recently, however, there have been publications indicating that the initial microbial population attaching to concrete in the marine environment changes with time to produce a common “generic” concrete biofilm [39,40,41]. Earlier results had indicated this possibility. Ref. [42] used DGGE analysis to identify the dominance of *Thiobacillus thiooxidans* and *Acidithiobacillus* sp. on corroded areas of concrete sewer pipes and Ref. [43], using metagenomic techniques on 175 samples from various surfaces of 9 piers along the Hong Kong coast, identified considerable differences between microbiomes on concrete and metal structures. Interestingly, they found that metal surfaces contained more functional genes involved in iron uptake, while those involved in iron regulation and storage were more common on concrete. Relatively few iron-oxidizing bacteria were found on either type of surface, however. The pier floors yielded higher Cyanobacteria-dominated microbiomes than the walls, consistent with floors wetted by wave action remaining damp for longer. Cyanobacteria rely on liquid water more than chlorophytes, which can use water vapor for growth. Table 1 shows the microorganisms that we have identified in the literature as attaching to concrete under continuous and intermittent seawater exposure conditions. The processes involved in the microbial biodeterioration of concrete structures in the marine environment are discussed in the following sections.

#### 2.2.1. Microbial Adhesion and Biofilm Development

Biodeterioration of concrete in the marine environment begins with microorganisms adhering to its surface, forming a biofilm. The alkaline nature of new concrete inhibits the activity of microorganisms [46], but the pH falls rapidly under the wetting and leaching influence of the marine environment, whose pH is generally between 7.5 and 9 [50], enabling microbial adhesion and metabolic activity [51]. Laboratory experiments demonstrated the effect of reducing the surface pH of concrete samples on the rate of microbial adhesion from seawater. The first colonizers are bacteria, which can themselves produce acid metabolites that further reduce pH and increase susceptibility of the concrete surface to microbial attack. The earliest studies concentrated on concrete corrosion in sewers by acid-producing bacteria [52], but more recent studies have determined the effects of concrete structure and composition on general bacterial adhesion (e.g., [40,51,53,54]). The effect of surface roughness of the substratum on biofilm formation has been shown to influence the types of bacteria initially adhering to immersed materials [54], although it has been suggested that this may not be very important for particles as small as bacteria adhering to concrete [55]. The presence of reinforcing rods within the concrete has a much greater effect than rugosity [40]. The last cited authors found that bacterial groups with increased adherence to reinforced concrete included members of the family *Magnetospiraceae* and the genera *Portibacter*, *Rubripirellula*, and *Rhodopirellula*; these are not associated with a particular requirement for iron, although *Magnetospiraceae* have been found in Fe–Mn deposits [56] and *Portibacter* have been found in iron slags in marine situations [57]. It is likely that metal ions from within the corroding concrete can reach the surface and become available to bacteria in the surrounding seawater. Iron is an important nutrient for bacterial cells and, as a component of certain natural stones, may act as an attractant for adhering microorganisms [58]. 

Ref. [40] found that *Ponticaulus* sp. and *Hyphomonas* sp. were two of the most abundant bacterial genera in early biofilms on concrete in seawater and suggested that they are pioneer organisms that are later outgrown by other genera. These two genera have been found to play important roles in biofilm formation on steel in the marine environment [59]. Ref. [39], after a series of experiments to assess the effects of different stone substrata (including concrete) on marine biofilm formation, similarly concluded that the biofilms converged over time to a generic marine type and that the underlying substrata did not play a significant role in community composition. Where the substrata are similar, as in this case where they were all types of natural or artificial stone, this may be true, but there are certainly differences between the biofilm-formers on very different substrates in seawater, such as concrete and wood, for example [60].

The initial attachment of cells to the concrete surface leads to the upregulation of bacterial genes that code for the production of extracellular polymeric substances (EPS), which strengthen the attachment and encourage the adhesion of circulating cells and other materials. EPS are diverse, including not only polysaccharides, the principal component, but also proteins, nucleic acids, and lipids [61]; their potential effects on concrete are varied and complex. Cactus polymer, for example, has been used in Mexico to improve the properties of Portland cement [62] and, along with bacterial EPS, to consolidate limestone surfaces [63]. Bacterial EPS can be used to improve the viscosity and cohesion of concrete [64]. On the other hand, the EPS-containing biofilm is a stable environment for microbial cells, enabling them to remain active and attached to the concrete surface and minimizing the loss of corrosive metabolites [65]. There is considerable information on EPS, including their make-up, production, and effects [61,66,67,68,69,70,71], and this will not be further detailed here. Their production plays a part in the development of a complex biofilm, which eventually leads to the adherence of higher organisms, the readily visible “marine biofouling”, sometimes called “hard fouling” because of the presence of shell-bearing animals such as barnacles and oysters; this final stage in the marine fouling process is not within the remit of the current article.

#### 2.2.2. Concrete Corroding Microorganisms and Mechanisms

Although the initial bacterial biofilm on seawater-immersed concrete may have little immediate effect on its structure, it encourages adhesion and growth of further microorganisms, many of which may have corrosive activity.

##### Organic Acid Producing Microorganisms

Many heterotrophic bacteria such as *Vibrio*, Acidobacteria, and *Bacillus* produce organic acids during their metabolism. Organic acids attack the concrete, causing decalcification of hydration products, which leads to higher inherent porosity and cracking [72]. They are also produced by fungi, although these filamentous microorganisms are less common in the marine environment. Indeed, Ref. [73] discussed whether fungi are, in fact, metabolically active in seawater, pointing out that many of them are parasitic on other living creatures. They emphasize that DNA sequencing techniques for fungi in general are underdeveloped, explaining, at least in part, why so few marine fungi have been described. Ref. [74] stated that most marine fungi are saprobes that rely on the high levels of organic matter generally found in coastal environments. Whether fungi in the open sea are potentially corrosive is debatable. Nevertheless, many terrestrial fungi produce organic acids and, if active in saltwater and sediments, will be capable of concrete deterioration.

Direct evidence of the fungal decay of concrete, then, is missing, although there is no doubt that these microorganisms have the metabolic capacity to produce corrosive compounds [75], and recent genomic and metabolomic analyses have demonstrated that at least one marine fungal species (*Emericellopsis cladophorae*, associated with marine algae) has the genetic capacity to produce carbohydrate-active enzymes under saline conditions [76]. Ref. [77] suggested that a *Fusarium* species isolated from degraded concrete along with *Thiobacillus* could be responsible for more rapid degradation than the bacteria. Ref. [78] reported a fungus (*Fusarium oxysporum*) as being responsible for the corrosion of three concrete bridges over the river Nile, but only the presence of the fungus was reported, and there was no direct evidence of its involvement in the corrosion process. Other studies have reported the isolation of microorganisms from corroded concrete that were deemed unlikely to be involved in biodeterioration (e.g., [79]). Nevertheless, there is some evidence that fungi, simply by their acid-producing metabolism, may be able to corrode concrete under certain conditions [80]. Ref. [81] selected three fungi previously shown to grow on concrete surfaces, although not underwater, to test a nanosilica coating as a protective layer on concrete. When incubated with control uncoated concrete blocks in a fungal growth medium, all caused weight loss after 3 months. The most destructive fungus was *Aspergillus tamarii*, a fungus isolated originally from historic buildings in Havana. Ref. [75] discuss in detail the potential of fungi to cause concrete corrosion, although they do not cite any definitive cases of such corrosion in the marine environment.

##### Inorganic Acid-Producing Bacteria

Autotrophs can produce inorganic (mineral) acids, which are more corrosive than organic acids. These include nitrifying bacteria, such as *Nitrosomonas* and *Nitrobacter*, which produce nitric acid [82,83], and sulfur-oxidizing bacteria (SOB) such as *Thiobacillus*, *Thiothrix*, *Thiomicrospira*, *Beggiatoa*, etc., [83,84]. The latter group produces sulfuric acid that reacts with concrete to form gypsum, which has poor structural properties. This is considered the main concrete corrosion process in sewers, often in conjunction with anaerobic, heterotrophic sulfate-reducing bacteria (SRB) such as those in the genera *Desulfovibrio* and *Desulfomicrobium*, which, under low oxygen conditions, reduce the sulfate in seawater to sulfides that can then be converted to corrosive sulfuric acids by the SOB [85]. SRB are mainly, but not exclusively, anaerobic bacteria and would be active in the marine environment either in sediments or beneath biofilms. Ref. [86] give a detailed description of SRB corrosion in concrete sewers.

Heterotrophic (organic carbon-utilizing) bacteria produce a variety of organic acids during metabolism and growth. A very wide variety of bacterial species with this ability are found in seawater and their concrete corroding ability cannot be ignored, although there is little published evidence relating to specific genera and species.

The potential, specifically corrosive nature of the microorganisms detected in immersed concrete biofilms is noted, where relevant, in Table 1.

### 2.3. Influence of Seawater Exposure Regime on Concrete Biofilm Formation

Oxygen is a critical component in determining the growth of microorganisms. Those found in well-oxygenated waters are very different from the facultative or obligate anaerobes that can adsorb to surfaces at lower depths. Our review of the literature has not yielded well-constructed experiments that allow direct comparisons of permanently and intermittently immersed concrete. The perfect set-up would be similar to that employed by Ref. [87], who studied the corrosion of a concrete beam exposed for 7 years to atmospheric, splash, tidal, and submerged zones around Qingdao Wheat Island, China. Unfortunately, they did not include biofilm formation in their study, although they did report that barnacles were only found in the tidal zone while oysters were found in the submerged zone. Comparison between these types of concrete immersion in seawater must rely, therefore, on published case histories, with no possibility of citing controlled experiments or even paired results.

#### 2.3.1. Concrete in the Submerged Zone

**Figure 1:**
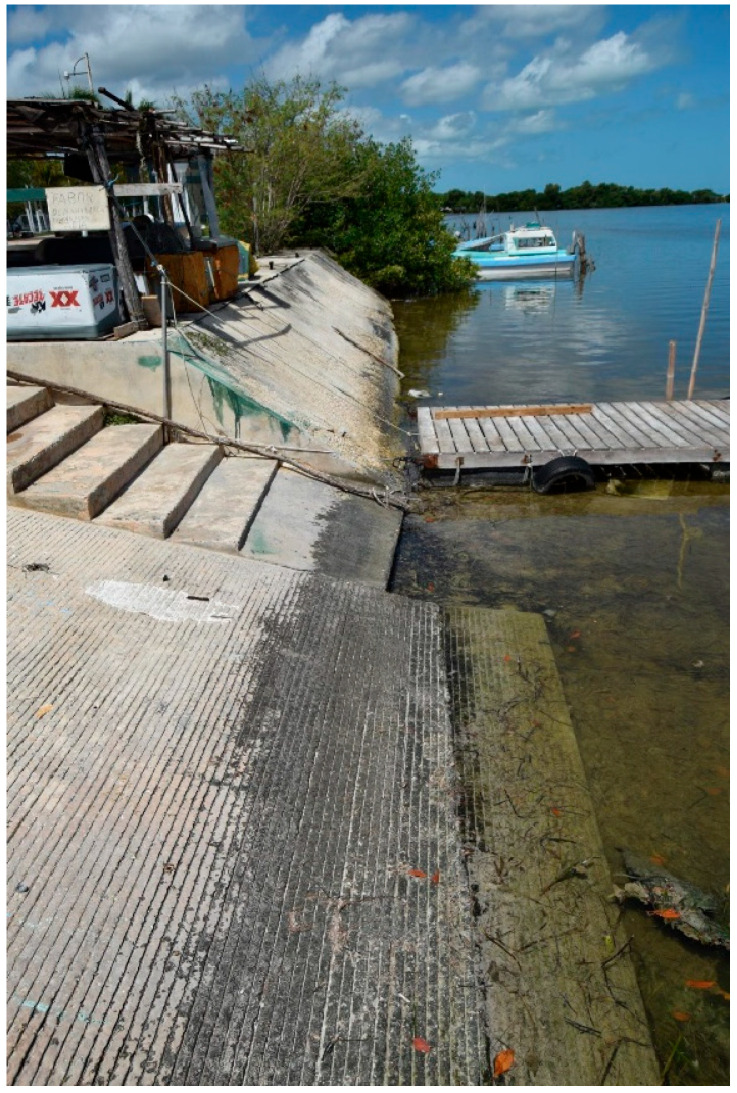
Concrete docks for artisanal fishing boats in Campeche, southern Mexico. Note that areas reached only by the marine spray appear devoid of (visible) microbial colonization, whereas intermittently submerged surfaces are covered by black biofilms. Permanently submerged areas are heavily covered by phototrophic communities. San Francisco de Campeche, Campeche, Mexico.

Permanently immersed concrete may avoid strong corrosion if the seawater is sufficiently low in oxygen, although microorganisms active at low oxygen levels may still produce aggressive compounds [45,47,88,89]. In less deep waters, sufficient oxygen may still be present to allow chemical corrosion and biological activity will certainly be present, with its associated dangers. Figure 1 shows a concrete slope in a small dock; the black biofilm in the splash zone is very different from the green, submerged zone film containing photosynthetic cyanobacteria and algae.

Apart from concrete structures such as oil storage tanks associated with offshore oil platforms, underwater tunnels, and foundations, this section also includes laboratory and in situ simulations aimed at determining the parameters involved in concrete biocorrosion, testing protective processes, and evaluating new types of concrete. Even when such experimental pieces are subjected to the real marine environment in situ, they are normally kept completely immersed for the required time (e.g., [90,91]). The laboratory tests almost always involve complete immersion of test pieces in natural or artificial seawater; they are often inoculated with single microorganisms and in this case, do not represent real conditions. Nevertheless, we are including in this article references to studies where conditions are sufficiently similar to those in vivo as examples of biodeterioration of permanently immersed concrete. 

Ref. [92], for example, used a laboratory system to demonstrate the effect of dissolved oxygen on concrete corrosion by sulfur-oxidizing bacteria added to seawater; lower dissolved oxygen levels resulted in reduced bacterial growth and less corrosion. 

Ref. [93], similarly in vitro, showed that the marine benthic diatom, *Cylindrotheca closterium*, was able to liberate and utilize silicon from a cementitious mortar. The diatom utilized the Si to produce new frustule material during cell division. Diatoms have often been shown to deposit on immersed concrete; indeed, [94] suggested that diatoms adhered to a recycled concrete surface could offer increased durability and demonstrate increased water resistance in laboratory experiments. 

Ref. [40] reported controlled experiments to determine the formation of biofilms on concrete of similar composition to that of the Oslofjord subsea tunnel, which had shown deterioration at sites where saline groundwater had intruded in previous investigations [95]. At this earlier time, the group had demonstrated the presence of ammonia- and nitrite-oxidizing microorganisms, in particular *Nitrosopumilus* sp., and iron-oxidizing bacteria within the *Mariprofundus* sp., as well as various heterotrophic bacteria and archaea at these tunnel sites. However, this microbial population will have included organisms from groundwater, as well as marine bacteria. In their later investigations [40], they incubated in water from the fjord concretes of similar composition to those in the tunnel. The fjord water was filtered (50 µm), kept in the dark, and recirculated to ensure oxygen saturation during incubation with the concrete samples, which were removed at intervals over 65 weeks. Bacteria and archaea in the concrete and seawater samples were subjected to DNA analysis. The microbial populations in the thin biofilms on the concrete were significantly different from those in the seawater, even though both populations were composed of typical marine genera and changed with time. Different concrete compositions showed different colonization patterns during the first weeks, but these differences tended to even out, suggesting a replacement of initially adhering microorganisms with more generic concrete colonizers, as also suggested by Ref. [39]. No special acid-producing genera were detected in the sessile populations, although calcium was removed from the concrete surfaces, indicating corrosion. However, metabolomic studies indicated that the mixed acid fermentation pathway was common in the biofilm.

Ref. [40]’s results on microorganisms present in the biofilms, along with results from other articles on seawater and rare but interesting freshwater situations are included in Table 1. 

#### 2.3.2. Concrete in the Splash and Tidal Zones

**Figure 2:**
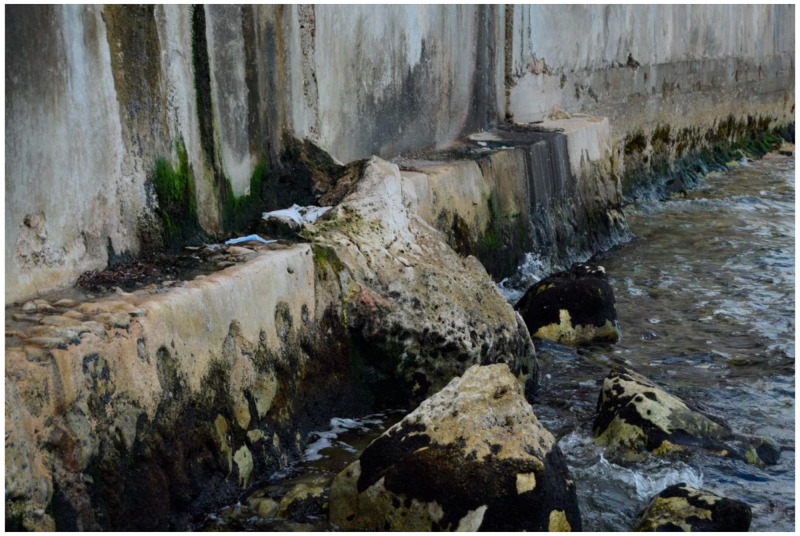
Intertidal concrete structures such as the base of this fisherman’s house, built over a rocky shore, exhibit a heterogeneous coverage of microorganisms. Lerma village, Campeche, Mexico.

**Figure 3:**
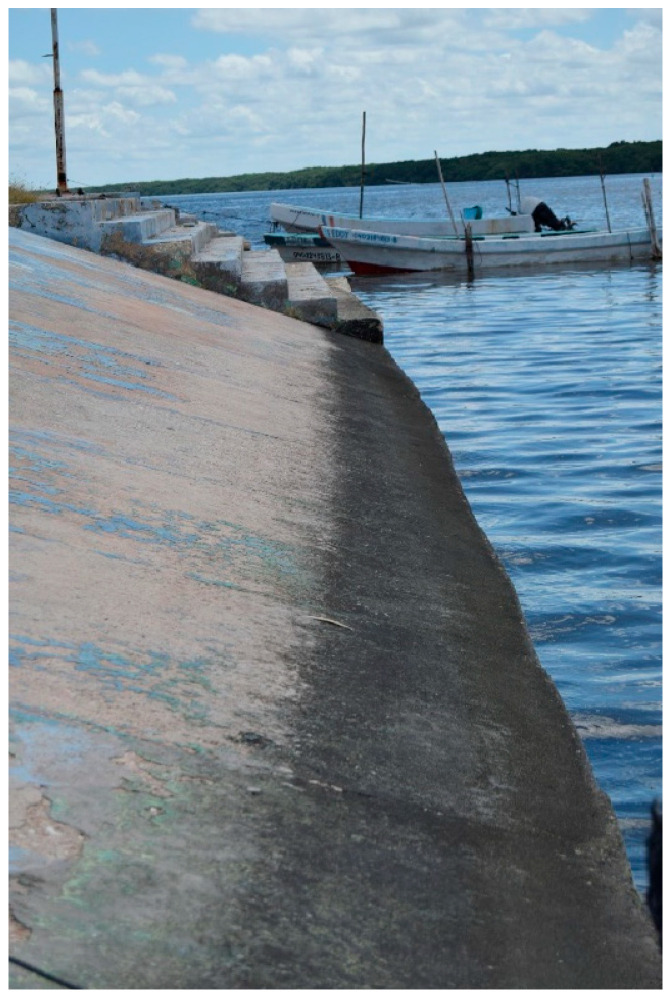
Biofilms covering concrete surfaces often exhibit a black phenotype, indicating the likely presence of scytonemin-producing cyanobacteria as a functional adaptation for water stress and excessive insolation. San Francisco de Campeche city, Campeche, Mexico.

Artificial shoreline structures such as piers, reefs, seawalls, embankments, and jetties are common in developed coastal regions and serve as examples of intermittently immersed concrete that are also, incidentally, often steeply inclined. Obviously, the more steeply inclined the concrete, the less time it will spend covered in water. As noted in the section on chemical concrete corrosion, the splash zone is most affected by the corrosive action of seawater constituents plus oxygen from the atmosphere. However, simple observation indicates that this is not the zone most affected by biodeterioration (Figure 2 and Figure 3).

Figure 2 demonstrates intense corrosion and biofilm formation on a vertical concrete structure in Campeche, Mexico.

The intertidal zone shows deep pits and cracks in the concrete, associated with dark biofilms, while the splash zone above demonstrates black/green biofilms but without the intense corrosion of the intertidal zone. It is possible that the microbiological colonization has protected this area from the normally more intense chemical corrosion that occurs in the splash zone. Alternatively, it may be the greater wave impact at the wall base that is at least partially responsible for greater physical degradation.

A similar, but less intense, situation can be seen in Figure 3. Here, corrosion (surface spalling) of the concrete in the more inclined splash zone can be seen, but the intertidal zone is covered by a dark gray/black biofilm with associated, relatively light, biodegradation.

Ref. [96] found that bacterial biofilms colonizing breakwaters along an island coast were dominated by Cyanobacteria, Proteobacteria, and Bacteroidetes. They were different from planktonic bacteria in the same locations, as shown by [40].

Although not normally in contact with seawater, reservoirs are associated with various concrete structures such as walls, gate piers, and slopes that are only intermittently immersed in water and could give an indication of concrete colonizers on intermittently immersed structures. Ref. [44] studied the bacterial communities at various points on four reservoirs in the Yangtze River basin using Miseq DNA sequencing. Proteobacteria, Cyanobacteria, and Chloroflexi were the major colonizers, with the most common groups being *Leptolyngbya*, Anaerolineaceae, and Polynuceobacter. The concrete gate piers had the highest proportion of sulfate-reducing bacteria and were considered to be at the highest risk, although ammonia oxidizers were also predominant.

A recent report on the presence of metagenomes associated with anammox (ammonium oxidizing) bacteria in water seepages on the concrete inner surface of the Subsea Oslofjord tunnel [97] is the first indication that these as-yet un-isolated anaerobes could be involved in deep sea concrete corrosion. Anammox bacteria are associated with biofilms that can lead to localized acidification [98]. Nitrifying bacterial markers (*Nitrosomonas*, *Nitrosopumilus*, *Nitrospirales*, and *Nitrospirota*) were also detected at the site. Once leaked into the tunnel, the higher oxygen environment (the equivalent of a splash zone) would allow the production of inorganic nitrogen acids, encouraging concrete corrosion.

## 3. Interactions between Chemistry and Microbiology in Concrete Degradation: Some Speculations

Although difficult to model, it is still fairly clear that certain ions in seawater lead to a reduction in the durability of concrete. These ions can be both utilized and produced by microorganisms and hence it is impossible to ignore their influence on so-called “abiotic” concrete corrosion in the oceans, although this will vary enormously, of course, depending on the microorganisms present and the surrounding physicochemical conditions. The effects of microbial adhesion and biofilm formation are themselves extremely variable, following the almost limitless variability of microorganisms. It is, however, possible to speculate on the possible interactions between chemical corrosion and microbial growth and activity.

The bacterial biofilm, with its accompanying EPS formed on the concrete surface, can confer a degree of protection on the substratum. EPS can maintain the hydration of the underlying concrete, even when seawater is not covering it. Hence the desiccation associated with tidal and splash zones would not occur and abiotic corrosion would be reduced. It has also been suggested that biofilm can block the entry of aggressive sulfate and chloride ions into the concrete structure, thus enhancing its durability [99]. Biofilm can physically impair the leaching of calcium hydroxide from the concrete [100], reducing ongoing corrosion. Nevertheless, there is no doubt that microbial activities can adversely affect concrete durability [101]; the infiltration of bacteria and fungi into concrete pores can lead not only to their blocking but also to chemical concrete deterioration by microbial activities such as acid production. 

The analysis of both the chemical and biological components of concrete biofilms from the marine environment would enable further potential interactions to be determined. Both qualitative and quantitative changes in cells and chemical compounds occur over the formation of the biofilm (e.g., [53,102,103,104,105]), but no detailed analyses of such changes in biofilm formation on concrete in the marine environment have been published.

## 4. Artificial Reefs: A Special Case

Although in use by mankind for thousands of years, artificial reefs are now being increasingly considered as possible answers to the diminishing biodiversity in coastal ecosystems [106,107,108,109,110,111]. They are not always made of concrete, natural rocks, or other materials often being used. However, Ref. [112] showed that there was no significant difference between the facultative marine fungi colonizing artificial reefs made of concrete or limestone sampled over 23 months in the Mississippi Gulf, although occasional phylotypes occurred on only one type of substrate. Overall, the concrete reef had the highest fungal diversity. A wide variety of fungi belonging to basidiomycetes, zygomycetes, and, principally, ascomycetes, were identified. 

Ref. [60] showed that permanently immersed concrete blocks had a much higher microbial diversity than wood, both being common artificial reef materials. These two substrates were suspended approximately 10 m below the surface, close to the sea floor in Shuangdao Bay, China, for up to 5 months, with weekly sampling when possible. Proteobacteria and Bacteroidetes were the major OTUs identified in both substrates. Cyanobacteria were dominant on concrete in the first 4 weeks but thereafter diminished in prevalence.

Ref. [113] used metagenomics to determine the bacteria present in marine benthic samples taken from beneath one- and ten-year-old concrete reefs in the Beibu Gulf, China. They found that Rickettsiales, Moraxellaceae, and *Acinetobacter* were enriched in 10-year-old samples, while *Francisella* was enriched in the one-year-old reef sediments. Unfortunately, the bacteria adhered to the concrete reefs were not sampled, although it might be assumed that some of these microorganisms would attach to buried parts of the reefs.

Ref. [41] used metagenomics to study the bacterial colonization of three types of cement for use in artificial reefs around the coral reefs off Weizhou Island, China, sampling over three concurrent seasons. Initially, Cyanobacteria dominated all the concrete samples, with Proteobacteria also common. The latter then began to overtake the phototrophic bacteria, especially in the standard concrete samples where Fusobacteriota also began to appear. The initial but non-permanent dominance of Cyanobacteria on immersed concrete has also been reported by [60]. Ref. [41] found that the standard, unmodified cement in artificial reefs showed lower biodiversity than the other two types of cement, which contained bioactive materials. The lower microbial diversity on its surface indicates the slightly toxic nature of this cement to marine bacteria, as previously shown for bacteria in groundwater stored in concrete or earthen ponds, the former being lower in diversity and abundance [114]. Indeed, concrete has been described as having especially deleterious consequences on biodiversity in all aquatic ecosystems [115]; this concords with research suggesting that a relatively mature microbial biofilm on concrete in the marine environment is somewhat generic, differing little from normal structural concrete composition [39,40,41].

## 5. Conclusions and Perspectives

The chemical corrosion of concrete in the marine environment is well understood in terms of the aggressive seawater ions and their interaction with concrete components. The long-term durability of coastal or deep-sea concrete structures, however, depends on a host of factors and it can be impossible to predict useful life simply based on the structural components. More detailed studies of different types of concrete and varied seawater constituents are necessary to enable useful predictions of concrete durability.

Although, in principle, it is possible to understand the theoretical basis of chemical concrete corrosion, its complexity has so far prevented us from efficiently predicting this process in the different types and zones of seawater around the world.

How much more difficult, then, is the understanding and prediction of microbial biodeterioration of concrete and the potential interactions between abiotic and microbial corrosion? Studies on “simple” colonization of concrete surfaces by marine microorganisms are hampered by a lack of suitable detection techniques. Nevertheless, new methods of study such as metagenomics and metabolomics are being developed and employed and will surely lead to a vast increase in our knowledge and understanding of the interactions between marine microorganisms and concrete. At the same time, surface protection methods, although not discussed in this article, are continually being developed. It is not necessary to understand how microbial cells attach to and attack concrete if effective methods of preventing this are available, although the purist will understand that a lack of knowledge of the reasons for any effective treatment can prejudice such treatment and its control. The acknowledgment that there is a need to conserve some concrete raw materials (such as sand) and that concrete itself is prejudicing the world’s biodiversity is already leading to changes in the way that we produce and monitor marine long-term structures. Concrete scientists and biologists will doubtless find themselves participating more frequently in future collaborations.

## Figures and Tables

**Table 1:** Some of the microorganisms detected in biofilms on permanently or intermittently immersed concrete.

Microorganism	Permanent	Intermittent	Reference	Comments
*Lyngbya*		+	[44]	Cyanobacteria Yangtze river reservoir
*Leptolyngbya*		+	[44]	Cyanobacteria Yangtze river reservoir
Cyanobacteria	+	+ +	[45] [41] [46]	Breakwater AR In situ concrete test
Proteobacteria		+	[45]	Breakwater
Bacteroidetes		+ +	[45] [46]	Breakwater In situ concrete test
*Anaerolinea*		+	[44]	Yangtze river reservoir
*Polynucleobacter*		+	[44]	Yangtze river reservoir
Sulfate reducers		+	[44]	Yangtze river reservoir
Ammonia oxidizers *Nitrosopumilus* sp.		+ +	[44] [47]	Yangtze river reservoir Ammonia-oxidizing archaea. Oslofjord undersea tunnel
Desulfobacteria Desulfobacterales		+ + +	[48] [46] [47]	Sulfate reducers In situ concrete test Sulfate reducers. Oslofjord undersea tunnel
Firmicutes		+ +	[48] [46]	Tidal areas. The phylum includes sulfur bacteria. May produce endospores In situ concrete test
Acidobacteria		+	[46]	In situ concrete test. Acid producers
*Chloroflexi*		+	[46]	In situ concrete test Heterophototrophic filamentous bacteria
*Nitrospina, Nitrospira*		+	[47]	Nitrite-oxidizing bacteria. Oslofjord undersea tunnel
*Nitrosomonas*		+	[47]	Anoxic ammonia oxidizers. Oslofjord undersea tunnel
*Scalindua*		+	[47]	Anammox bacteria. Oslofjord undersea tunnel
*Mariprofundus*		+	[47]	Stalked iron-oxidising bacteria. Oslofjord undersea tunnel
*Ponticaulus*	+		[40]	Archaea In vitro study
*Hyphomonas*	+		[40]	Stalked bacteria. In vitro study
Planctomycetales	+ +	+	[40] [41] [46]	In vitro study. Budding bacteria AR In situ concrete test
Rhodobacterales	+		[40]	Primary marine surface colonizers. In vitro study
Caulobacteriales	+		[40]	In vitro study. Stalked bacteria
*Portibacter*	+		[40]	In vitro study. Bacteroidetes.
*Bacillus* (Firmicutes)	+		[49]	Bridge
*Brachybacterium*	+		[49]	Bridge
*Flavobacterium*	+		[49]	Bridge
*Lysinibacillus*	+		[49]	Bridge
*Thiomonas perometabolis*	+		[49]	Bridge. Sulfur oxidizer
*Propiogenium*	+		[41]	AR Anaerobe
*Vibrio*	+		[41]	AR
*Clostridium*	+		[41]	AR Anaerobe
Fusobacteria	+		[41]	AR Anaerobes
Actinobacteria	+	+	[41] [46]	AR In situ concrete test

AR = artificial reef.

## Data Availability

No new data were generated during the writing of this article.
